# Effect of functional knee loading on articular cartilage MRI T2 relaxation time and thickness in patients at risk for knee osteoarthritis

**DOI:** 10.1016/j.ostima.2024.100173

**Published:** 2024-01-26

**Authors:** Hayden F. Atkinson, Trevor B. Birmingham, Codie A. Primeau, Anthony A. Gatti, Rebecca F. Moyer, Jaques S. Milner, David W. Holdsworth, J. Robert Giffin

**Affiliations:** a Faculty of Health Sciences, Western University, London, ON, Canada; b London Health Sciences Centre, London, ON, Canada; c Bone & Joint Institute, Western University, London, ON, Canada; d Arthritis Research Canada, Vancouver, BC, Canada; e Department of Physical Therapy, University of British Columbia, Vancouver, BC, Canada; f Department of Radiology, Stanford University, Palo Alto, CA, USA; g NeuralSeg Ltd., Hamilton, ON, Canada; h Faculty of Health, Dalhousie University, Halifax, NS, Canada; i Robarts Research Institute, Western University, London, ON, Canada; j Schulich School of Medicine & Dentistry, Western University, London, ON, Canada

**Keywords:** Articular cartilage, Exercise, Osteoarthritis, MRI, T2 relaxation time

## Abstract

**Objectives:**

The objectives of this study were: 1) to evaluate the effect of a functional loading stimulus on MRI-acquired T2 relaxation time (T2) and thickness of knee articular cartilage, and 2) to compare the response between patients at risk for knee OA and healthy controls.

**Design:**

A total of 32 participants (16 healthy controls [24.7 ± 3.0 years], and 16 at-risk participants [37.5 ± 12.2]) underwent 3T MRI T2 mapping scans immediately before and after a standardized 25-minute functional loading stimulus on a computerized treadmill that included a variety of challenging walking conditions. Groups were defined using the Osteoarthritis Initiative Control (healthy) and Incidence Cohort (at-risk) Criteria. We analyzed changes in T2 between groups in the superficial and deep layers of tibiofemoral, patellar, and trochlear cartilage, and for tibiofemoral cartilage thickness using multivariate linear mixed-effects models.

**Results:**

T2 was shorter in the superficial cartilage layers in both groups. The mean combined change (95 % confidence interval) in T2 of the superficial layer was -3.80 ms (-4.87; -2.73) for at-risk participants and -3.89 ms (-4.96; -2.82) for healthy controls. The between-group difference in change was 0.09 ms (-1.04; 1.22). There was a decrease in articular cartilage thickness in the lateral compartment for healthy controls (-0.14 mm [-0.24; -0.04]), otherwise there were no changes detected.

**Conclusions:**

Consistently shorter T2 was observed in the articular cartilage of patients at risk for knee OA and in healthy controls, after a challenging walking test, but with no concurrent change in cartilage thickness, suggesting a similar articular cartilage response to functional loading.

## Introduction

Degradation of articular cartilage is characteristic of osteoarthritis (OA), affecting joint structures, and leading to substantial pain and disability[1]. Articular cartilage plays a crucial role in dissipation of loads, enabling nearly frictionless joint motion[2]. Cyclic loading is essential for exchange of nutrients and metabolites between articular cartilage and synovial fluid[3]. However, excessive or insufficient loading can contribute to articular cartilage damage, affecting its mechanical function, leading to the onset and progression of OA[4].

Magnetic resonance imaging (MRI) allows for visualization and quantification of articular cartilage structure and biochemistry. Compositional T2 relaxation mapping of articular cartilage provides a non-invasive surrogate measure for articular cartilage integrity, distinguishing patients with knee OA[5], or at risk for knee OA, from healthy controls[6]. Changes in T2 relaxation appear prior to radiographic features of OA and are associated with onset and progression of such features[7]. At-risk patients often demonstrate prolonged T2 relaxation time, indicating collagen disorganization, increased free water concentration, and compromised extracellular matrix[8], which are precursors to morphological changes[9].

In vivo studies have used T2 mapping to assess the mechanical response of articular cartilage to joint loading, including controlled and pragmatic approaches such as the application of controlled loads in the scanner[10], or completing T2 mapping scans before and after activities such as walking[11], running[12], squatting[13], and cycling[14]. These studies consistently report acute shortening of articular cartilage T2 relaxation time, particularly in load-bearing compartments. This is attributed to fluid egress from the cartilage into the joint space, especially in the more permeable superficial layer[15]. Recovery to baseline values typically occurs between 30 min to several hours[16], suggesting the intensity of activity does not increase the risk for developing OA in healthy individuals[12]. With regards to articular cartilage thickness, chronic changes are associated with trauma and imbalance in load[17], while acute changes are minimal with level walking in healthy controls[18]. Increased BMI[19], reduced gait speed[20], and ACL injury[21,22] may increase acute cartilage strain. These changes are likely transient, and generally smaller than those induced by running in healthy controls[12]. As a result, consensus on clinically important acute changes for cartilage morphology and composition has yet to be established.

Previous studies in this domain have typically assessed healthy controls. However, in established knee OA, the difference in T2 relaxation response to static load bearing is greater than that of healthy controls[10]. This suggests OA not only erodes articular cartilage, but also compromises its resilience. The earliest stage at which this critical difference occurs, however, is unknown, as are the duration and intensity of load required to evoke this response. Identifying this inflection point may provide the opportunity for early and personalized treatment in at-risk individuals at a stage where intervention can yield lifelong benefits[23]. Moreover, at-risk individuals are more likely to participate in loading activities without realizing that they may be harmful. Understanding load-induced compositional and morphological changes of articular cartilage in at-risk individuals can provide insight on disease mechanisms and help establish safe loading parameters for this population.

Therefore, the objectives of this study were: 1) to evaluate the effect of a standardized functional loading stimulus on MRI-acquired T2 relaxation time and thickness of knee articular cartilage, and 2) to compare the response between patients at risk for knee OA and healthy controls. We hypothesized that 1) functional loading will shorten T2 relaxation time and reduce articular cartilage thickness across all knee compartments, and 2) at-risk patients will demonstrate larger changes in these measures compared to healthy controls. Within the at-risk group, we expect ACL-injured patients to demonstrate larger changes.

## Methods

### Participants

A total of 32 participants (16 at risk, 16 controls) were recruited based on 80 % power to detect a change of moderate effect size (0.5) from pre- to post-loading (two-sided alpha = 0.05)[24]. At-risk patients were recruited from the on-campus Fowler Kennedy Sport Medicine Clinic at Western University. Healthy controls were recruited via response to on-campus advertisement at Western University. Eligibility criteria for at-risk patients were based on the Osteoarthritis Initiative (OAI) Incidence Cohort criteria[25], which include any one of the following: knee symptoms (pain, aching, stiffness) in the past 12 months; a history of knee injury affecting gait for two weeks or more; or a history of knee surgery. For healthy controls, eligibility criteria were also based on the OAI control cohort, which includes freedom from symptoms such as pain, aching, stiffness in the past year, and no history of knee surgery, or injury that would have affected the ability to walk normally for more than two weeks. We opted to recruit healthy participants between 18 and 30 years of age to limit the chances of age-related changes in articular cartilage composition in this reference sample[26], and adjusted for age in the statistical analysis. We matched the sex and body mass index (BMI) of the patients due to their effects on knee joint loading[27,28]. All participants completed the Knee injury and Osteoarthritis Outcome Score (KOOS), with higher scores indicating greater function and decreased pain[29]. All participants provided written informed consent to participate in the study, approved by the institution's Research Ethics Board.

### Testing protocol

**Fig. 1:**
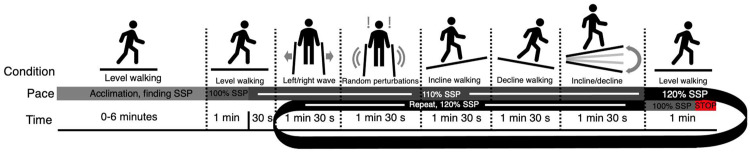
Timeline of the functional loading stimulus, including all conditions encountered by the participants. The shaded bar progresses sequentially through the functional walking tasks and is color-coded based on walking speed at each phase of the activity. Left/right wave consists of the treadmill translating 30 cm laterally, once per second. Random perturbations consist of belt slips and stops, rapid pitches (10${}^{\circ }$), and rapid translations (30 cm). Incline or decline walking consists of walking at a sustained grade of ±10${}^{\circ }$ Incline/decline consists of the treadmill alternating in pitch from ±10${}^{\circ }$ every 2 s. SSP = self-selected pace.

Participants completed a 3T MRI scan before and after a standardized dynamic functional loading stimulus that consisted of 25 min of challenged walking under a variety of conditions (Fig. 1). Testing was performed in the morning to minimize potential effects of load incurred throughout the day. Participants agreed to avoid high-impact and high-intensity activities for two days prior to the test date. Between scans participants walked the same 780 m route from the biomechanics laboratory to the MRI suite at their preferred pace, accompanied by the tester.

### Imaging

We scanned the more affected knee of at-risk patients, identified by risk factors, history of previous injury and/or current symptoms (meeting OAI Incidence Cohort criteria). We scanned the right knee of healthy controls. We used a 3T Siemens Magnetom Trio magnet and 15-channel Siemens PRISMA knee coil, located in the Centre for Functional and Metabolic Mapping, Robarts Research Institute, Western University. Total scan time was 30 min. The entire protocol is reported in Table 2. For all sequences, the center of the field of view was positioned at the center of the joint space, with the posterior borders of the volume positioned parallel to the posterior aspects of the femoral condyles to minimize medial-to-lateral obliquity in the sagittal direction. Participants were seated 30 min prior to the pre-loading scan to reduce the effect of loading incurred earlier in the morning. For the pre-loading scan, the T2 mapping sequence was performed last, resulting in a total of at least 45 min of unloading before applying the T2 mapping sequence, sufficient time for articular cartilage to recover from imposed strain[16]. For the post-loading scan, the T2 mapping sequence was performed first to avoid dissipation of the effect of the functional loading stimulus.

### Image processing

#### Relaxation time

**Fig. 2:**
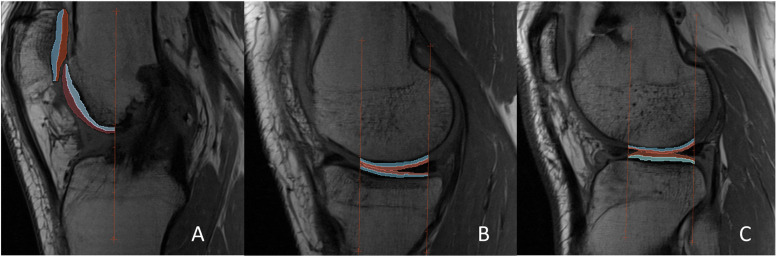
Sagittal view of the atlas-based segmentation technique, as well as application of the laminar splitting into superficial (red-shaded) and deep zones (blue-shaded) for the a) patellofemoral compartment, b) medial compartment, and c) lateral compartment.

We generated T2 relaxation maps using in-house software by fitting T2-weighted image intensity pixel-by-pixel to the equation S(TE) ∝ PD exp(−TE/T2), where TE is the echo time, PD is the proton density, and S(TE) is signal intensity, using a Levenberg-Marquardt mono-exponential fitting algorithm implemented with Insight Toolkit[30]. The first echo was excluded from the decay curve to eliminate the effect of stimulated echoes[31]. We used the first echo images for segmentation, as they provided the highest signal-to-noise ratio, and made identification of chemical shift artifacts more apparent than later echoes, allowing us to better exclude voxels affected by chemical shift from the ROI. Images were manually segmented by an imaging scientist (8 years of musculoskeletal image segmentation experience, trained by a musculoskeletal radiologist and rheumatologist) who was blinded to timepoint. Three consecutive slices were manually segmented for each load-bearing region of the medial femur, medial tibia, lateral femur, lateral tibia, patella, and trochlea with a standardized anatomical atlas[32] using 3D Slicer software[33]. The load-bearing region for tibiofemoral cartilage was defined as the central portion (from anterior-to-posterior) as identified by the anatomical atlas. In the sagittal direction, the slice including the distal apex of the medial and lateral femur in the extended position served to define the central slice for the load-bearing medial and lateral compartments. For the patella and trochlea, the three central slices of the patella were used for segmentation. Following segmentation, we applied a script to automatically divide ROIs into superficial and deep laminar layers, based on the minimum Euclidean distance to the nearest pixel, ensuring that the border between laminae was equidistant between the respective articular surface and bone-cartilage interface. Fig. 2 demonstrates the segmentation technique, including the atlas used to define the region of interest in the anterior-posterior direction, as well as an example of medial, lateral, and patellofemoral cartilage segmentations, with the laminar splits applied to create superficial and deep subregions.

#### Articular cartilage thickness

An identical segmentation technique was applied for measuring articular cartilage thickness in the tibiofemoral compartments, using a higher resolution 3D DESS sequence. Images and ROIs from the T2 mapping segmentation were anatomically registered to the 3D DESS images to identify the same articular cartilage. Seven slices, rather than three, were used for articular cartilage thickness measures to account for the difference in slice thickness between sequences.

#### Functional loading stimulus

Following the baseline scan, all participants completed the functional loading stimulus in the Wolf Orthopaedic Biomechanics Lab, Fowler Kennedy Sport Medicine Clinic, Western University. The loading stimulus consisted of 25 minutes of challenged walking on the Gait Real-time Analysis Interactive Laboratory, a computerized treadmill capable of moving with six degrees of freedom within a virtual reality environment, consisting of an interactive landscape projected onto a 180-degree wraparound screen in front of the participant, that responded in accordance with the speed, position, and orientation of the treadmill (MotekforceLink, Amsterdam, NL). The functional loading stimulus was pre-programmed and standardized to be identical and repeatable between participants, apart from self-selection of baseline walking speed. Participants self-selected their walking speed over the course of the first 2 min, starting at 1.00 m/s and incrementally increasing by 0.01 m/s every 5 s until the participant indicated they had attained their normal walking speed. Following a 6-minute acclimation period[34], we subjected participants to changes in speed (110–120 % baseline speed), inclines and declines (±10°), lateral sways (1 m/s, 15 cm left/right), and random pre-specified perturbations in the form of rapid belt slips, sagittal plane pitches, and frontal plane sways. Participants wore a passive ceiling-mounted harness for safety purposes, designed only to provide support in case of a fall. Participants also reported their rating of perceived exertion on the 6 to 20 Borg Scale. A full timeline of the functional loading stimulus is provided in Fig. 1.

### Statistical analysis

For the primary analysis, a composite measure for T2 relaxation time in the entirety of the superficial and deep layers of the knee articular cartilage was derived using the mean of the segmented load-bearing regions (Fig. 2). T2 relaxation times for at-risk and control groups were compared before and after loading using multivariate linear mixed-effects models (StataCorp LLC, College Station, TX). Separate models were built for superficial and deep layers, incorporating group (control vs. at-risk) and time (pre- vs. post-loading) as binary variables with an interaction term, and random intercepts per participant. The same model was used for the secondary analysis, applied individually to each laminar layer for all segmented regions. Within- and between-group differences were assessed using pairwise comparisons, adjusted for multiple comparisons with Sidak correction. Results were reported as adjusted mean T2 relaxation time for both timepoints, including within-group, between-group, and load-induced changes across the sample, with 95 % confidence intervals (CI). Articular cartilage thickness was analyzed using the same model as the secondary analysis (region-specific), but without laminar splitting. We conducted post-hoc sensitivity analyses including controlling for age and BMI. Analyses were then repeated when splitting the at-risk group into individuals with and without prior ACL rupture. For these analyses, participants were categorized as either 1) healthy controls, 2) at-risk with ACL rupture, or 3) at-risk without ACL rupture. All analyses were performed using a two-sided p-value of <0.05 to indicate statistical significance.

## Results

### Participants

Participant characteristics are summarized in Table 1. Of the patients at risk at risk for knee OA 7 had ACL ruptures, 6 had frequent symptoms, 2 had meniscal tears, and one had an articular cartilage lesion. Participants rated the functional loading stimulus as 13.2 ± 2.2 on the Borg scale, equivalent to moderate-intensity exercise[35]. All participants completed the study without incident or adverse events.

**Table 1:** Participant characteristics.

	Healthy Controls (*n* = 16)	At Risk for OA (*n* = 16)
**Age**, *years*	24.7 ± 3.0	37.5 ± 12.2
**Sex**, *no (%)*		
Male	13 (81)	13 (81)
**Body mass index**, *kg/m^2^*	24.3 ± 3.2	26.7 ± 3.0
**Knee injury and Osteoarthritis Outcome Score**^a^**,***scored 0 to 100*		
Pain	98.4 ± 2.7	80.0 ± 12.8
Other symptoms	96.5 ± 4.2	78.2 ± 9.6
Function in daily living	99.6 ± 0.8	89.9 ± 12.5
Function in sport and recreation	96.9 ± 4.8	70.0 ± 21.5
Knee-related quality of life	98.5 ± 2.7	58.3 ± 17.7
**Baseline Walking Speed,***(m/s)*	1.15 ± 0.06	1.15 ± 0.10
**Rating of perceived exertion,***(6–20 Borg Scale)*	11.7 ± 1.9	11.9 ± 1.5

*Values are reported as means with standard deviations unless otherwise specified.

a KOOS = Knee injury and Osteoarthritis Outcome Score – 0 indicates extreme knee symptoms; 100 indicates no knee symptoms. *Abbreviation*: OA = osteoarthritis

**Table 2:** MRI Protocol.

Sequence	TR (ms)	TE/SLT (ms)	FOV (mm)	Matrix Size	Slices	Slice Thickness (mm)	Slice Gap (mm)	Bandwidth (Hz)	Acceleration Parameters	Averages	Variables
Sagittal 3D DESS	16.3	4.7	140	384×307	160	0.7	–	–	–	1	Cartilage thickness
Sagittal 2D GRE T1rho Mapping	6.7	3.4/0, 10, 40, 80	140	256×256	32	3.0	0.6	500	–	2	–*
Sagittal 2D MESE T2 Mapping	2700	11.1, 22.2, 33.3, 44.4, 55.5, 66.6, 77.7	120	384×269	31	3.0	0.5	–	GRAPPA PE Accel. factor: 2 PE Ref. lines: 24	1	T2 relaxation time

TR = repetition time, TE = echo time, SLT = spin lock time, FOV = field of view, B1 = radiofrequency field, ms = milliseconds, 3D = three dimensional, DESS = dual-echo steady state, 2D = two dimensional, GRE = gradient echo, MESE = multi-echo spin echo, GRAPPA = Generalized Autocalibrating Partial Parallel Acquisition.

Order of acquisition following loading was as follows, to prioritize time-sensitive changes in T2 relaxation time after the functional loading stimulus: 1) Sagittal T2 Mapping, 2) Sagittal 3D DESS, 3) Sagittal T1rho Mapping.

⁎ *The T1rho Mapping sequence was not used for outcome measures of this study. Images were acquired for sequence optimization testing, while serving the dual-purpose of providing additional unloading time prior to T2 mapping acquisition.*

### T2 relaxation time response to loading

#### Primary analysis (Composite superficial and deep cartilage regions)

**Fig. 3:**
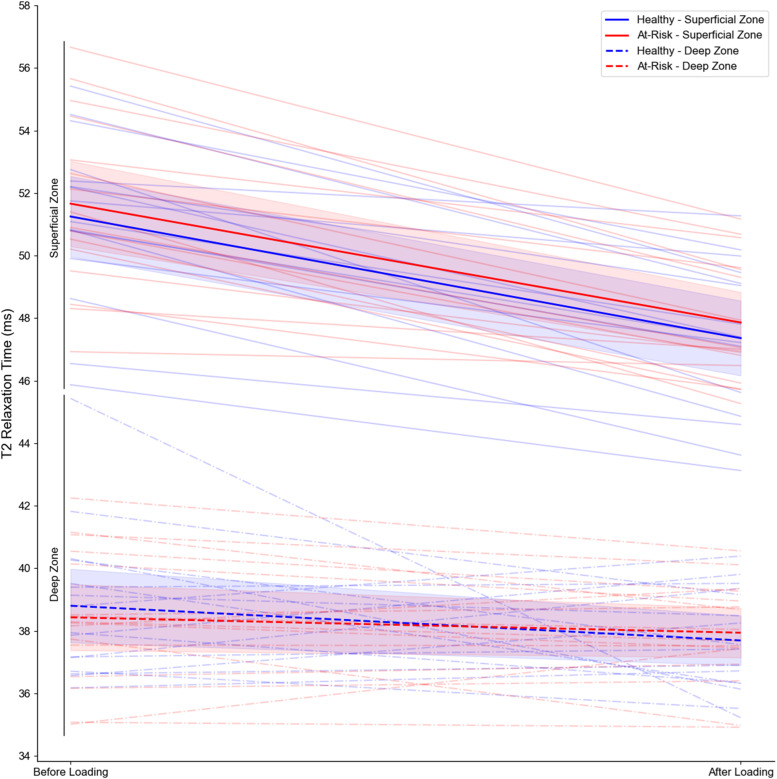
Changes in composite superficial (top, solid lines) and composite deep (bottom, dashed lines) articular cartilage T2 relaxation time in patients at risk for knee osteoarthritis (*n* = 16; red) and healthy controls (*n* = 16; blue) before and after completing a functional loading stimulus. Group means (with shaded 95 % confidence intervals) are presented as bolded lines, and individual participant responses are presented as translucent lines to demonstrate the variability in response.

Composite T2 relaxation time of the superficial and deep layers of knee articular cartilage before and after loading for both groups are illustrated in Fig. 3. For the superficial layer, T2 relaxation times shortened for both groups after the functional loading stimulus, and the change between groups was similar. Specifically, the mean change (95 %CI) in T2 relaxation time of the superficial layer was −3.80 ms (−4.87; −2.73) for at-risk patients and −3.89 ms (−4.96; −2.82) for healthy controls. The between-group difference in change was 0.09 ms (−1.04; 1.22). For the composite T2 relaxation shortening of the deep layer, the change was smaller and remained similar between groups after loading. Specifically, T2 relaxation time shortened by −0.49 ms (−2.01; 1.03) for patients at risk for OA and −1.11 ms (−2.63; 0.41) for healthy controls. The between-group difference in change was 0.62 ms (−0.98; 2.22). Results were similar while also controlling for age and BMI.

#### Secondary analysis (Region-Specific)

Results for the superficial and deep layers of each region are summarized in Table 3. Results were consistent when the mixed-effects regression analyses were repeated for each superficial layer of each segmented region of articular cartilage. Apart from the superficial trochlear compartment and the patellar compartment (but only in the healthy group), there were statistically significant within-group changes for all regions, indicating that T2 relaxation times shortened following the functional loading stimulus in both groups. There were, however, no significant differences between groups in the stimulus-induced change. In the deep layers of the articular cartilage, there were no significant changes within groups or differences between groups in the stimulus-induced change. Results were similar when controlled for age and BMI.

**Table 3:** Unadjusted changes in T2 relaxation time in superficial and deep regions of the knee articular cartilage before and after a loading stimulus in individuals considered at risk for developing knee osteoarthritis (*n* = 16) and healthy controls (*n* = 16).

		Superficial T2 relaxation time (in ms)			Deep T2 relaxation time (in ms)		
Zones	Group	Pre-loading	Post-loading	Within Change	Pre-loading	Post-loading	Within Change
**Medial Femur**	*Healthy*	55.75 (54.10 to 57.39)	51.42 (49.78 to 53.06)	**−4.32** **(−5.61 to −3.04)**	40.57 (38.77 to 42.37)	38.37 (36.56 to 40.17)	−2.20 (−4.60 to 0.20)
	*At-Risk*	54.80 (53.16 to 56.45)	49.36 (47.70 to 51.01)	**−5.44** **(−7.56 to −3.33)**	39.70 (37.89 to 41.50)	38.09 (36.28 to 39.89)	−1.61 (−4.01 to 0.79)
	**Between Difference**	−0.94 (−4.06 to 2.17)	−2.06 (−5.18 to 1.06)	−1.12 (−3.60 to 1.36)	−0.87 (−4.30 to 2.55)	−0.28 (−3.70 to 3.14)	0.59 (−1.94 to 3.12)
**Medial Tibia**	*Healthy*	50.94 (49.46 to 52.42)	45.91 (44.44 to 47.39)	**−5.03** **(−6.84 to −3.21)**	39.14 (37.81 to 40.48)	38.19 (36.85 to 39.52)	−0.96 (−2.82 to 0.91)
	*At-Risk*	52.47 (51.00 to 53.95)	47.32 (45.84 to 48.79)	**−5.16** **(−6.97 to −3.34)**	38.81 (37.48 to 40.14)	38.66 (37.32 to 40.00)	−0.15 (−2.02 to 1.71)
	**Between Difference**	1.53 (−1.27 to 4.34)	−1.40 (−1.40 to 4.21)	−0.13 (−2.04 to 1.78)	−0.34 (−2.87 to 2.19)	0.47 (−2.06 to 3.00)	0.81 (−1.16 to 2.77)
**Lateral Femur**	*Healthy*	53.01 (51.60 to 54.40)	49.66 (48.26 to 51.06)	**−3.34** **(−4.56 to −2.12)**	39.73 (38.39 to 41.06)	38.69 (37.35 to 40.03)	−1.04 (−2.91 to 0.83)
	*At-Risk*	53.35 (51.94 to 54.75)	50.79 (49.39 to 52.19)	**−2.55** **(−3.77 to −1.33)**	39.20 (37.86 to 40.54)	38.32 (36.98 to 39.65)	−0.88 (−2.75 to 0.99)
	**Between Difference**	0.34 (−2.32 to 3.00)	1.13 (−1.53 to 3.79)	0.79 (−0.49 to 2.07)	−0.53 (−3.06 to 2.01)	−0.37 (−2.91 to 2.17)	0.15 (−1.82 to 2.12)
**Lateral Tibia**	*Healthy*	45.30 (43.53 to 47.08)	42.44 (40.66 to 44.22)	**−2.86** **(−4.21 to −1.51)**	35.76 (34.05 to 37.47)	35.52 (33.81 to 37.23)	−0.25 (−2.07 to 1.58)
	*At-Risk*	46.01 (44.24 to 47.79)	43.97 (42.19 to 45.75)	**−2.05** **(−3.40 to −0.69)**	36.01 (34.30 to 37.72)	36.69 (34.98 to 38.40)	0.68 (−1.15 to 2.50)
	**Between Difference**	0.71 (−2.66 to 4.08)	1.53 (−1.84 to 4.90)	0.82 (−0.61 to 2.24)	0.25 (−3.00 to 3.50)	1.17 (−2.08 to 4.42)	0.93 (−1.00 to 2.85)
**Patella**	*Healthy*	49.03 (46.85 to 51.21)	46.88 (44.70 to 49.06)	−2.15 (−4.46 to 0.16)	38.81 (37.05 to 40.56)	38.16 (36.41 to 39.92)	−0.64 (−2.73 to 1.44)
	*At-Risk*	51.69 (49.51 to 53.87)	48.82 (46.64 to 51.00)	**−2.86** **(−5.18 to −0.55)**	40.31 (38.56 to 42.06)	40.33 (38.58 to 42.09)	0.02 (−1.16 to 2.10)
	**Between Difference**	2.65 (−1.48 to 6.79)	1.94 (−2.19 to 6.08)	−0.71 (3.15 to 1.73)	1.50 (−1.83 to 4.83)	2.17 (−1.16 to 5.50)	0.67 (−1.52 to 2.86)
**Trochlea**	*Healthy*	50.88 (49.00 to 52.76)	49.14 (47.25 to 51.02)	−1.75 (−3.86 to 0.36)	46.65 (44.76 to 48.54)	45.98 (44.09 to 47.87)	−0.68 (−2.42 to 1.07)
	*At-Risk*	53.39 (51.51 to 55.27)	52.93 (51.05 to 54.81)	−0.46 (−2.57 to 1.65)	48.39 (46.50 to 50.29)	47.76 (45.87 to 49.65)	−0.63 (−2.38 to 1.11)
	**Between Difference**	2.51 (−1.06 to 6.08)	**3.80** **(0.22 to 7.37)**	1.29 (−0.94 to 3.51)	1.74 (−1.85 to 5.33)	1.79 (−1.80 to 5.38)	0.05 (−1.79 to 1.88)

Mixed effects regression model. Analyses were also adjusted with Sidak correction for multiple comparisons.

Values were similar when also controlling for age and body mass index as covariates.

**Bolded** estimates represent statistically significant associations at the 5 % level.

### Sensitivity analyses of ACL-Injured patients

Characteristics used to categorize participants as healthy controls (*n* = 16), at-risk with ACL rupture (*n* = 7), and at-risk without ACL rupture (*n* = 9) are summarized in Supplemental Table 1, and results for comparisons of the superficial and deep layers of each region are summarized in Supplemental Table 2. Individuals in the at-risk but without ACL rupture group were older than the other groups.

T2 relaxation time for the composite superficial and deep layers of knee articular cartilage for patients at risk with ACL rupture, at risk without ACL rupture, and healthy controls before and after loading are illustrated in Supplemental Figure 1. Overall findings were similar when comparing healthy controls to both at-risk groups, regardless of ACL injury status, with no statistically significant differences in T2 relaxation times between groups for the composite superficial or deep measures. However, individuals in the at-risk with no ACL rupture experienced significantly greater shortening in T2 relaxation time for the composite superficial measure compared to those in the at-risk with ACL rupture group, with a between group difference of −1.61 ms (−3.12; −0.11). Changes in the composite deep layer were similar across all three groups.

When evaluating specific subregions, T2 relaxation time was statistically significantly shorter in the superficial medial femur for the at-risk without ACL rupture group compared to both the healthy controls (−3.53 ms [−5.99; −1.07]) and to those at-risk with ACL rupture (−5.51 ms [−8.48; −2.54]). Additionally, shortening of T2 relaxation time was significantly greater in the medial tibia for those in the at-risk without ACL rupture group compared to those with ACL rupture (−2.80 ms [−5.34; −0.25]). Changes in all other subregions for the superficial and deep layers were similar among all three groups.

### Articular cartilage thickness response to loading

Changes in cartilage thickness are summarized in Table 4. As with T2 relaxation time, results were consistent when the analyses were repeated when controlled for age and BMI in each compartment. The only significant change was observed in the lateral tibia of the healthy control group (−0.14 mm [−0.24; −0.04]), in comparison to a change of only −0.01 mm (−0.11; 0.09) in the at-risk group, yielding the only significant difference in cartilage thickness change (0.13 mm [0.02; 0.23]) between groups. There were no other statistically significant changes within or between groups for any other compartments, indicating that functional loading stimulus had a minimal effect on cartilage thickness. The articular cartilage of the at-risk group was slightly thicker at baseline in all compartments, except for the medial femur, which was slightly thinner, although none of the differences reached statistical significance. Results were similar after controlling for age and BMI at all timepoints.

**Table 4:** Unadjusted changes in articular cartilage thickness for each tibiofemoral region of interest before and after a loading stimulus in individuals considered at risk for developing knee osteoarthritis (*n* = 16) and healthy controls (*n* = 16).

Zones	Group	Pre-Loading Thickness (mm)	Post-Loading Thickness (mm)	Within-Group Change (mm)
**Medial Femur**	*Healthy*	2.04 (1.80 to 2.27)	2.01 (1.77 to 2.24)	−0.03 (−0.12 to 0.06)
	*At-Risk*	1.88 (1.64 to 2.11)	1.83 (1.60 to 2.07)	−0.04 (−0.13 to 0.04)
	**Between-Group Difference**	−0.16 (−0.61 to 0.29)	−0.17 (−0.62 to 0.28)	−0.01 (−0.11 to 0.08)
**Medial Tibia**	*Healthy*	1.75 (1.59 to 1.92)	1.70 (1.54 to 1.86)	−0.05 (−0.13 to 0.03)
	*At-Risk*	1.89 (1.73 to 2.06)	1.82 (1.66 to 1.98)	−0.08 (−0.15 to 0.003)
	**Between-Group Difference**	0.14 (−0.17 to 0.45)	0.12 (−0.19 to 0.42)	−0.02 (−0.11 to 0.06)
**Lateral Femur**	*Healthy*	1.86 (1.64 to 2.08)	1.81 (1.59 to 2.03)	−0.05 (−0.12 to 0.02)
	*At-Risk*	1.93 (1.71 to 2.15)	1.92 (1.71 to 2.15)	−0.01 (−0.08 to 0.07)
	**Between-Group Difference**	0.07 (−0.35 to 0.49)	0.11 (−0.31 to 0.53)	0.04 (−0.04 to 0.12)
**Lateral Tibia**	*Healthy*	2.62 (2.35 to 2.88)	2.48 (2.21 to 2.75)	**−0.13 (−0.24 to −0.04)**
	*At-Risk*	2.86 (2.60 to 3.13)	2.85 (2.59 to 3.12)	−0.01 (−0.11 to 0.09)
	**Between-Group Difference**	0.25 (−0.26 to 0.75)	0.37 (−0.13 to 0.87)	**0.13 (0.02 to 0.23)**

## Discussion

Consistent with our hypothesis, we observed a shortening in T2 relaxation times across all superficial regions of the knee articular cartilage, as well as the deep region of the medial femur after a standardized functional loading stimulus. Previous research suggests that the superficial compartments are more responsive to the load due to the greater potential for fluid exchange at the articular surface given increased permeability[15]. The deep compartment of the medial femur was the only deep region with significant changes, likely due to higher medial knee loads during ambulation[36].

We hypothesized that the knee articular cartilage of patients at risk for knee OA would experience a greater response to loading compared to the healthy controls, because longitudinal changes in compositional MRI appear to be a marker for earlier cartilage damage after knee trauma[37]. Contrary to our hypothesis, the at-risk and healthy control groups demonstrated similar T2 relaxation time responses across all subregions following the loading stimulus. This finding suggests that the overall response of knee articular cartilage to the present challenging and functional knee loading paradigm is similar between healthy controls and those at risk for knee OA (Table 2), indicating that moderate-intensity weight-bearing activities are likely safe for individuals at risk for knee OA. This finding aligns with a systematic review using quantitative MRI in patients at-risk for or with knee OA, which concluded that longer-term exercise, involving repeated bouts of exercise comparable in intensity to our functional loading stimulus, do not result in articular cartilage damage[38].

Contrary to our hypothesis, our post-hoc subgroup analyses (Supplemental Table 2) showed that patients with a history of ACL had less shortening of T2 relaxation time in the superficial cartilage of the medial compartment than the rest of the at-risk group. This may be related to differences in medial-lateral load distribution dependent on risk factors[36].

Although the at-risk patients generally had thicker cartilage than healthy controls at baseline, differences were not statistically significant, except for the medial femur. This may be an example of “cartilage swelling” that has been observed after joint injury[39]. The loading stimulus led to decreases in cartilage thickness between 0.5 % to 5.3 % across all compartments in the sample, with most changes in thickness being on the lower end. The only significant change was in the lateral tibia of the healthy control group (Table 4). The comparable morphologic response among groups provides additional support for the similarities of articular cartilage following functional loading. Our findings contrast with those in the acutely ACL-deficient sample of Crook et al.[21], but are comparable with those with the ACL-reconstructed sample of Pamukoff et al.[22]. Both studies used the uninjured contralateral limb as a control, and measured cartilage strain after 20[21] or 30[22] minutes of level walking. The ACL-deficient sample demonstrated increased strain in the medial femoral and lateral tibial cartilage, while the ACL-reconstructed sample showed no within- or between-limb differences, apart from a small but significant decrease in the medial compartment of the uninjured limb. They also observed increased baseline cartilage thickness in the ACL-reconstructed limb compared to the uninjured limb, similar to our results. Taken together with our findings, this suggests that despite changes in cartilage composition and morphology due to joint trauma, cartilage function may recover over time[40].

This study has limitations. Although 32 participants provided adequate power to detect within-group changes, we may have lacked the power to detect differences among groups. The confidence intervals should be kept in mind when interpreting these results.. The magnitude of acute or longitudinal change in T2 relaxation that is clinically important is presently unknown. Conservatively, it should be acknowledged that the extremes of the 95 %CI for the mean differences for some ROIs may include important differences. For example, in the superficial medial femur, the lower end of the 95 %CI includes −3.8 ms greater shortening in T2 relaxation time for the at-risk group (Table 3). The present sample also consisted of mostly men, although the sex ratios were consistent between groups. While previous research has not detected sex-based relationships between cartilage composition and walking loads in healthy controls[41], more research is required. Furthermore, the at-risk group was nearly 12 years older than the control group, potentially introducing age as a confounder given the known age-dependent relationship in articular cartilage T2 relaxation time. This relationship, however, is typically only evident after age 45[26], whereas our sample is representative of a population without age-dependent changes in articular cartilage, which was reflected in our results. Our study focused exclusively on articular cartilage. Other tissues, such as the menisci, ligaments, musculature, and bone likely influence articular cartilage loading[42]. Integrative modeling approaches could provide greater insight on these relationships. Additionally, a voxel-based approach rather than a region-based approach may improve the ability to identify focal changes in T2 relaxation time and thickness[43]. We also did not evaluate gait biomechanics during the functional loading stimulus, nor did we model forces across the knee during the loading. Patients at risk for knee OA may have walked in a way that reduced the load on their knees (e.g., as a response to pain); however, most research suggests greater loads in patients with and at risk for knee OA[44]. Notably, the walking speeds and rate of perceived exertion were similar between groups, with identical loading stimuli and duration. Lastly, although we used accepted criteria to define control and at-risk groups, future research may also investigate larger, more homogenous samples of patients who share the same risk factors for incident knee OA.

Strengths of this study include its focus on patients at risk for knee OA, rather than those with established disease, and the use of a challenging, repeatable, and standardized loading stimulus that mimics a range of physiological movements and loads on the knee. We thoroughly analyzed the responses of both articular cartilage composition and thickness using multivariate linear mixed-effects models and adjusted for multiple comparisons. We completed several sensitivity analyses to control for potential covariates including age, BMI, and ACL status.

Repetitive microtrauma due to long-term aberrant loading is thought to contribute to OA, while an appropriate volume of weight-bearing activity is known to be protective of OA progression[45]. This suggests a “Goldilocks zone” of optimal loading to preserve articular cartilage structure and function, although the parameters of this zone are unknown[46]. Identifying protective and harmful loading thresholds should lead to improved strategies to manage OA, especially for those with existing risk factors[9]. Given the similar findings among the study groups, we suggest that the threshold for harmful loading is likely higher than applied in this study. This is encouraging for those at risk for knee OA and may help identify load tolerance thresholds for patients with risk factors for knee OA.

In conclusion, we observed inconsistently shorter T2 relaxation times in superficial knee articular cartilage following moderate-intensity joint loading, in line with our hypothesis. However, we did not observe concurrent changes in articular cartilage thickness for most compartments, and there were few differences between at-risk and healthy controls. The injured ACL subgroup demonstrated similar or smaller changes compared to the remainder of the at-risk group, contrary to our hypothesis. Although future work is required, the present results suggest that functional knee loading as part of a challenging walking test causes similar acute changes in both composition and thickness articular cartilage in patients at risk for knee OA and in healthy controls.

## Author contributions statement

All authors contributed to the research design, drafting and revision of the manuscript, and approval of the final version.

## Appendix Supplementary materials

Image, application 1
