# Supplementary material for: Effect of functional knee loading on articular cartilage MRI T2 relaxation time and thickness in patients at risk for knee osteoarthritis
Source: Osteoarthr Imaging. 2024 Jan 26;4(1):100173. doi: 10.1016/j.ostima.2024.100173 (PMC13228713; doi:10.1016/j.ostima.2024.100173)
Supplement: Supplementary file 1 [file mmc1.docx]

| **Supplemental Table 1**. Participant characteristics after dividing the at risk for osteoarthritis group into those who had an anterior cruciate ligament rupture, and those who are at risk for reasons unrelated to anterior cruciate ligament. | | | |
| --- | --- | --- | --- |
|  | **Healthy Controls (n=16)** | **At Risk for OA – ACL rupture (n=7)** | **At Risk for OA –**  **No ACL rupture (n=9)** |
| **Age**, *years* | 24.7 ± 3.0 | 28.3 ± 9.0 | 44.7 ± 9.3 |
| **Sex**, *no (%)* |  |  |  |
| Male | 13 (81) | 6 (86) | 7 (78) |
| **Body mass index**, *kg/m^2^* | 24.3 ± 3.2 | 25.9 ± 1.8 | 25.5 ± 3.8 |
| **Knee injury and Osteoarthritis Outcome Score** *^a^***,** *scored 0 to 100* |  |  |  |
| Pain | 98.4 ± 2.7 | 82.1 ± 13.2 | 78.3 ± 13.1 |
| Other symptoms | 96.5 ± 4.2 | 78.7 ± 8.1 | 77.8 ± 11.1 |
| Function in daily living | 99.6 ± 0.8 | 91.3 ± 13.1 | 88.9 ± 2.7 |
| Function in sport and recreation | 96.9 ± 4.8 | 70.0 ± 25.2 | 70.0 ± 19.8 |
| Knee-related quality of life | 98.5 ± 2.7 | 60.0 ± 21.5 | 57.0 ± 15.5 |
| **Baseline Walking Speed,** *(m/s)* | 1.15 ± 0.06 | 1.16 ± 0.12 | 1.14 ± 0.05 |
| **Rating of perceived exertion,** *(6-20 Borg Scale)* | 11.7 ± 1.9 | 11.5 ± 1.6 | 12.7 ± 1.2 |
| *Values are reported as means with standard deviations unless otherwise specified  ^a^ KOOS = Knee injury and Osteoarthritis Outcome Score – 0 indicates extreme knee symptoms; 100 indicates no knee symptoms.  *Abbreviations*: ACL = anterior cruciate ligament, OA = osteoarthritis | | |  |

| **Supplemental Table 2.** Unadjusted changes in T2 relaxation time in superficial and deep regions of the knee articular cartilage before and after a loading stimulus in individuals considered at risk for developing knee osteoarthritis with a history of anterior cruciate ligament injury (n=7), at-risk without a history of ligament injury (n=9), and healthy controls (n=16). | | | | | | | | | |
| --- | --- | --- | --- | --- | --- | --- | --- | --- | --- |
|  |  | **Superficial T2 relaxation time (in ms)** | | | | **Deep T2 relaxation time (in ms)** | | | |
| **Zones** | **Group** | **Within-Group Change** | **Between-Group Difference*** | | | **Within-Group Change** | **Between-Group Difference*** | | |
|  |  |  | *Healthy* | *At-risk, ACL rupture* | *At-risk, No rupture* |  | *Healthy* | *At-risk, ACL rupture* | *At-risk, No rupture* |
| **Medial Femur** | *Healthy* | **-4.33**  **(-6.53 to -2.12)** | -- | 1.98  (-0.69 to 4.65) | **-3.53**  **(-5.99 to -1.07)** | -2.20  (-4.87 to 0.46) | -- | 1.00  (-2.23 to 3.25) | 0.27  (-2.70 to 3.25) |
|  | *At-risk, ACL rupture* | -2.34  (-5.67 to 0.99) | 1.98  (-0.69 to 4.65) | -- | **-5.51**  **(-8.48 to -2.54)** | -1.20  (-5.23 to 2.83) | 1.00  (-2.23 to 3.25) | -- | -0.73  (-4.33 to 2.87) |
|  | *At-risk, No ACL rupture* | **-7.86**  **(-10.79 to -4.92)** | **-3.53**  **(-5.99 to -1.07)** | **-5.51**  **(-8.48 to -2.54)** | -- | -1.93  (-5.48 to 1.62) | 0.27  (-2.70 to 3.25) | -0.73  (-4.33 to 2.87) | -- |
| **Medial Tibia** | *Healthy* | **-5.03**  **(-6.91 to -3.14)** | -- | 1.44  (-0.84 to 3.73) | -1.35  (-3.46 to 0.75) | -0.96  (-3.03 to 1.12) | -- | 0.88  (-1.64 to 3.39) | 0.75  (-1.56 to 3.07) |
|  | *At-risk, ACL rupture* | **-3.58**  **(-6.44 to -0.73)** | 1.44  (-0.84 to 3.73) | -- | **-2.80**  **(-5.34 to -0.25)** | -0.08  (-3.22 to 3.06) | 0.88  (-1.64 to 3.39) | -- | -0.12  (-2.92 to 2.68) |
|  | *At-risk, No ACL rupture* | **-6.38**  **(-8.90 to -3.87)** | -1.35  (-3.46 to 0.75) | **-2.80**  **(-5.34 to -0.25)** | -- | -0.20  (-2.97 to 2.56) | 0.75  (-1.56 to 3.07) | -0.12  (-2.92 to 2.68) | -- |
| **Lateral Femur** | *Healthy* | **-3.34**  **(-4.70 to -1.99)** | -- | 0.58  (-1.07 to 2.22) | 0.96  (-0.55 to 2.46) | -1.04  (-3.09 to 1.02) | -- | -0.58  (-3.07 to 1.92) | 0.72  (-1.57 to 3.01) |
|  | *At-risk, ACL rupture* | **-2.77**  **(-4.81 to -0.72)** | 0.58  (-1.07 to 2.22) | -- | 0.38  (-1.45 to 2.20) | -1.61  (-4.72 to 1.49) | -0.58  (-3.07 to 1.92) | -- | 1.30  (-1.47 to 4.07) |
|  | *At-risk, No rupture* | **-2.39**  **(-4.19 to -0.58)** | 0.96  (-0.55 to 2.46) | 0.38  (-1.45 to 2.20) | -- | -0.31  (-3.05 to 2.42) | 0.72  (-1.57 to 3.01) | 1.30  (-1.47 to 4.07) | -- |
| **Lateral Tibia** | *Healthy* | **-2.86**  **(-4.32 to -1.41)** | -- | -0.02  (-1.78 to 1.74) | 1.47  (-0.16 to 3.09) | -0.25  (-2.27 to 1.78) | -- | 0.70  (-1.76 to 3.16) | 1.10  (-1.17 to 3,36) |
|  | *At-risk, ACL rupture* | **-2.88**  **(-5.08 to -0.68)** | -0.02  (-1.78 to 1.74) | -- | 1.49  (-0.48 to 3.45) | 0.45  (-2.61 to 3.52) | 0.70  (-1.76 to 3.16) | -- | 0.40  (-2.34 to 3.14) |
|  | *At-risk, No ACL rupture* | -1.40  (-3.33 to 0.54) | 1.47  (-0.16 to 3.09) | 1.49  (-0.48 to 3.45) | -- | 0.85  (-1.85 to 3.56) | 1.10  (-1.17 to 3,36) | 0.40  (-2.34 to 3.14) | -- |
| **Patella** | *Healthy* | -2.15  (-4.71 to 0.41) | -- | -1.36  (-4.46 to 1.75) | -0.21  (-3.07 to 2.64) | -0.64  (-2.96 to 1.67) | -- | 0.39  (-2.41 to 3.20) | 0.88  (-1.70 to 3.46) |
|  | *At-risk, ACL rupture* | -3.51  (-7.37 to 0.36) | -1.36  (-4.46 to 1.75) | -- | 1.14  (-2.31 to 4.60) | -0.25  (-3.75 to 3.24) | 0.39  (-2.41 to 3.20) | -- | 0.49  (-2.63 to 3.61) |
|  | *At-risk, No ACL rupture* | -2.36  (-5.76 to 1.05) | -0.21  (-3.07 to 2.64) | 1.14  (-2.31 to 4.60) | -- | 0.24  (-2.85 to 3.32) | 0.88  (-1.70 to 3.46) | 0.49  (-2.63 to 3.61) | -- |
| **Trochlea** | *Healthy* | -1.75  (-4.09 to 0.60) | -- | 1.06  (-1.79 to 3.90) | 1.46  (-1.15 to 4.08) | -0.68  (-2.61 to 1.26) | -- | -0.29  (-2.64 to 2.06) | 0.31  (-1.85 to 2.46) |
|  | *At-risk, ACL rupture* | -0.69  (-4.23 to 2.86) | 1.06  (-1.79 to 3.90) | -- | 0.41  (-2.76 to 3.57) | -0.97  (-3.89 to 1.95) | -0.29  (-2.64 to 2.06) | -- | 0.60  (-2.01 to 3.21) |
|  | *At-risk, No ACL rupture* | -0.28  (-3.41 to 2.85) | 1.46  (-1.15 to 4.08) | 0.41  (-2.76 to 3.57) | -- | -0.37  (-2.95 to 2.21) | 0.31  (-1.85 to 2.46) | 0.60  (-2.01 to 3.21) | -- |
| Mixed effects regression model. Analyses were also adjusted with Sidak correction for multiple comparisons.  Values were similar when also controlling for age and body mass index as covariates.  **Bolded** estimates represent statistically significant associations at the 5% level.  *The reference category for between-group comparisons was the healthy control (vs. either at-risk group), except for comparison between the two at-risk groups where at-risk of developing knee osteoarthritis with a history of anterior cruciate ligament injury was considered the reference category (vs. no ligament injury). | | | | | | | | | |

**
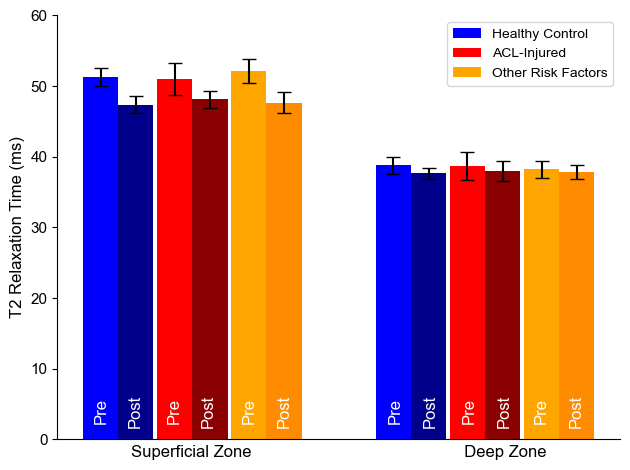
**

**Supplemental Figure 1.** Composite pre- and post-loading T2 relaxation time for the superficial (left) and deep (right) articular cartilage in healthy controls (n=16; blue), at-risk patients with ACL rupture (n=7; red), and at-risk patients without ACL rupture (n=9; orange), before and after completing the functional loading stimulus. Error bars represent 95% confidence intervals for the group mean.
